# Proposed Orbital Products for Positioning Using Mega-Constellation LEO Satellites

**DOI:** 10.3390/s20205806

**Published:** 2020-10-14

**Authors:** Kan Wang, Ahmed El-Mowafy

**Affiliations:** School of Earth and Planetary Sciences, Curtin University, Perth 6102, Australia; A.El-Mowafy@curtin.edu.au

**Keywords:** LEO, POD, orbit prediction, mega-constellation, positioning

## Abstract

With thousands of low Earth orbit (LEO) satellites to be launched in the near future, LEO mega-constellations are supposed to significantly change the positioning and navigation service for ground users. The goal of this contribution is to suggest and discuss the feasibility of possible procedures to generate the LEO orbital products at two accuracy levels to facilitate different positioning methods—i.e., Level A orbits with meter-level accuracy as LEO-specific broadcast ephemeris, and Level B orbits with an accuracy of centimeters as polynomial corrections based on Level A orbits. Real data of the LEO satellite GRACE FO-1 are used for analyzing the error budgets. For the Level A products, compared to the orbital user range errors (OUREs) of a few centimeters introduced by the ephemeris fitting, it was found that the orbital prediction errors play the dominant role in the total error budget—i.e., at around 0.1, 0.2 and 1 m for prediction intervals of 1, 2 and 6 h, respectively. For the Level B products, the predicted orbits within a short period of up to 60 s have an OURE of a few centimeters, while the polynomial fitting OUREs can be reduced by a few millimeters when increasing the polynomial degree from one to two.

## 1. Introduction

Global navigation satellite systems (GNSSs) currently provide positioning, navigation and timing (PNT) services to users in different areas. In certain challenging environments, GNSS signals may suffer from limited or even no visibility—e.g., in urban canyons. The signals from mega-constellation low Earth orbit (LEO) satellites, currently in developement with satellites continuously being launched, are nowadays becoming attractive for navigation purposes. Since these LEO satellites are primarily used for communications, to enable positioning, it is proposed that either additional payloads are added to these satellites to provide GNSS-like signals or employ their signals as signals of opportunity for positioning (SOOP) [1]. Different from the GNSS satellites that are located at the medium Earth orbits (MEOs), the LEO satellites at lower latitudes of about 300 to 1500 km [2] are able to provide ground users with much stronger signals, which enables positioning in GNSS-challenging areas and makes the signals more resilient against jamming [3]. A large number of LEO satellites launched or to be launched in the near future by companies such as Iridium, Globalstar, SpaceX, OneWeb, Samsung and Boeing does not only benefit the overall satellite geometry and thus the position dilution of precision (PDOP), the fast-moving LEO satellites and the rapidly changing geometry can also reduce the long convergence time in the precise point positioning (PPP) [4].

The concept of using LEO satellites for positioning is similar to that using GNSSs, which requires knowledge of the precise LEO satellite positions. The two-line-elements (TLEs) of the LEO satellites are published and updated daily by the North American Aerospace Defense Command (NORAD) [5]. Their provided accuracy of several kilometers, however, cannot fulfil the requirement for high-precision positioning. Similar to the precise orbit determination (POD) procedure for the GNSS satellites, one could rely on a ground network receiving the LEO signals to determine their orbits. Attempts have been made by TRANSIT, and a 3D root mean square (RMS) of the orbital errors of several meters was achieved using four ground stations [6,7]. The orbital accuracy improves when increasing the network density—i.e., with the root mean square error (RMSE) reduced to the sub-meter to meter-level in the radial, along-track and cross-track directions using 15 ground stations [8]. Benefiting from the GNSS receivers equipped onboard the LEO satellites, the signals from the higher GNSS satellites can be used for positioning the LEO satellites as users, and high-accuracy orbit determination becomes possible. Combining proper dynamic models with the GNSS observations in the reduced-dynamic orbit determination scheme, the onboard real-time LEO POD can achieve an accuracy of a few decimeters even when using the inaccurate GNSS broadcast ephemeris and considering the limited computational power available onboard [9]. Using precise GNSS products and comprehensive dynamic models in the post-processing mode, the reduced-dynamic LEO orbits can typically reach an accuracy of centimeters [10]. 

For different positioning techniques, the requirements on the orbital accuracy are different. For the single point positioning (SPP) using the pseudorange observations and the real-time kinematic (RTK) positioning with the orbital errors largely reduced by forming between-satellite differences, an orbital accuracy at meter-level could be sufficient. For PPP, however, which would benefit from the rapidly varying LEO satellite geometry, high-accuracy orbit at a few centimeters is the key to successful positioning. In this study, making use of the GNSS observations collected onboard the LEO satellites, and taking the needs of different methods (SPP, RTK and PPP) into consideration, the orbital products are proposed to be generated with two different levels of accuracy. At Level A, low-accuracy orbits are broadcast from LEO satellites to users in the form of ephemeris parameters. At Level B, high-accuracy orbital corrections with a high sampling rate would be transmitted from the ground processing center to users with low-order polynomials through Internet links.

Compared to the GNSS satellites, the LEO satellites experience more complicated dynamics due to their lower altitudes and, as a result, suffer from higher influences of the Earth’s gravitational field and the air drag [11]. As shown in [3], using the same set of the ephemeris parameters as the Global positioning system (GPS) legacy navigation (LNAV) message, an orbital user range error (OURE) induced by orbital fitting amounts to about 7 cm for the GPS satellites within a fitting interval of 4 h. For LEO satellites, however, this is only achievable within a much shorter fitting interval—i.e., from about 10 to 20 min. Additional and transformed ephemeris parameters are reported to be helpful to improve the OUREs [11]. The ephemeris parameters, however, need to be fit to the predicted but not the precise orbits. As discussed in [12], even based on good dynamic models, the prediction errors dramatically grow with the age of data (AOD) to several decimeters after one hour. The prediction interval that is closely related to the data uploading rate serves as the limiting factor for the OUREs. In this contribution, for Level A orbital products, the error budgets are analyzed in detail for the LEO broadcast ephemeris using real data of a typical LEO satellite, GRACE FO-1, which are subject to relatively strong influences of the Earth’s gravity field and the air drag. The study includes the infrastructure design, the analysis of the potential data gaps between subsequent satellite contacts, the prediction errors introduced by different dynamic models and for different prediction intervals, as well as the fitting errors of the ephemeris parameters at different fitting and prediction times. The detailed error budget is given for the Level A products in terms of OUREs and the 3D RMSE.

Similar to diverse GNSS real-time correction streams, e.g., provided by the International GNSS Service (IGS) real-time service (RTS) [13,14], precise Level B orbital corrections are computed based on the Level A products in this contribution and are supposed to be delivered to users via Internet links. The precise orbits can be extrapolated for tens of seconds corresponding to the latency between the reference time of the applied corrections and the time of processing [14]. The differences between the extrapolated orbits and the Level A orbits are then fitted with polynomials of different degrees. The polynomial fitting is analyzed at different prediction and ephemeris fitting times of the Level A orbits, as different orbital characteristics may apply at different phases of the Level A orbits. The detailed error budgets of the Level B products are given, and the benefits of increasing the polynomial degrees are discussed.

In general, the overall objective of this work is to design and discuss the feasibility of possible procedures to generate the LEO orbital products at two accuracy levels—i.e., less accurate Level A products as a broadcast ephemeris type, and high-accuracy Level B products as corrections to broadcast ephemeris that can be transmitted via the Internet. The paper starts with the proposed infrastructure design and the processing procedures of products A and B. This is followed by the analysis of different error types of these two orbital products using real LEO data. The results are discussed and conclusions are given at the end.

## 2. Processing Procedures

It is assumed that dual-frequency GPS observations on L1 and L2 can be continuously collected and stored onboard, downlinked to the ground monitoring stations (GMSs) during their ground contacts and transferred to the master processing center (MPC). The high-accuracy reduced-dynamic orbits can then be estimated and predicted in the MPC using comprehensive dynamic models and real-time precise GNSS products. In this section, the processing procedure is distinguished between Level A orbital products with a relatively low accuracy of sub-meters to meters and Level B orbital products with a high accuracy of centimeters. 

### 2.1. Level A Products

**Figure 1:**
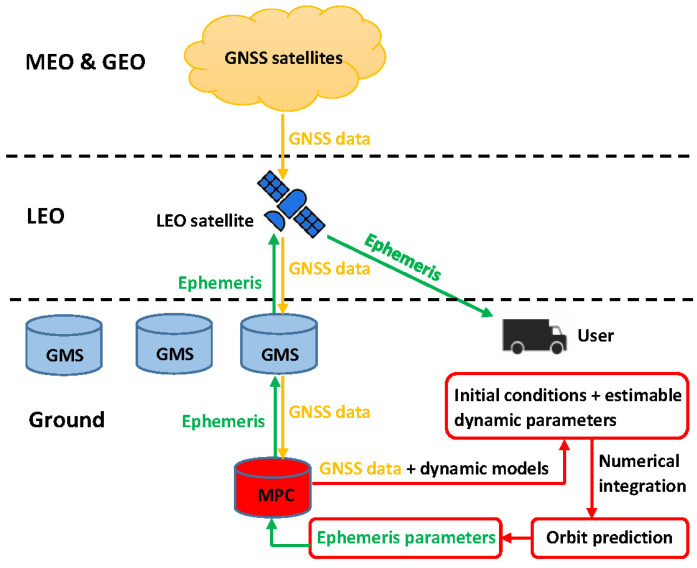
The processing procedure of the Level A orbital products.

The proposed Level A orbital products can be produced in the following steps, and the procedure is shown in Figure 1:

- The GNSS data collected onboard the LEO satellites are downlinked to the GMSs with a time interval ∆*T* between subsequent downloads, and then transferred to the MPC.
- High-accuracy reduced-dynamic orbits are then processed with comprehensive dynamic models (as will be described in Section 2.1.2), having the Keplerian elements at the initial condition and certain dynamic parameters estimated to compensate for the deficiencies in the dynamic models.
- The orbits are then predicted for several hours into the future with numerical integration, which will be discussed in Section 2.1.1. The prediction interval should cover at least a period of ∆*T* + ∆*t_p_*, where ∆*t_p_* is the time needed for the data downloading, processing and uploading during the next GMS–LEO contact.
- Next, LEO-specific ephemeris parameters (Section 2.1.3) are estimated using the least-squares adjustment to describe the predicted orbits within a pre-defined fitting interval ∆*t_F_* (Section 2.1.3), where ∆*t_F_* is normally selected much shorter than the prediction interval.
- The fitted ephemeris parameters are updated with an interval of ∆*t_U_* < ∆*t_F_*, so that overlapping time exists between two subsequent sets of ephemeris parameters.
- All the estimated ephemeris parameters are then uplinked to the LEO satellites, and the LEO satellites broadcast their ephemeris parameters to the users.

#### 2.1.1. Prediction Interval

**Figure 2:**
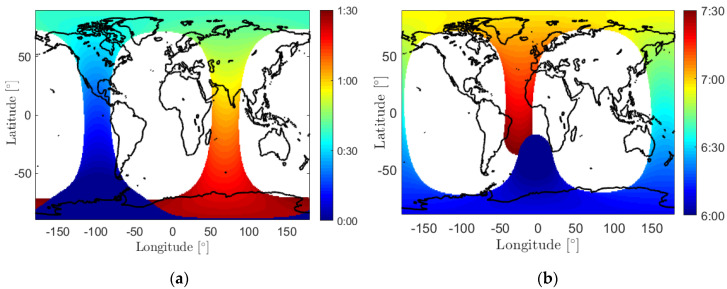
Ascending time of GRACE FO-1 for the day 14 August 2018, (**a**) from 0:00 to 1:30 in GPS time (GPST) and (**b**) from 6:00 to 7:30 in GPST. The almost equal colors around the North and the South Poles are caused by the spherical shape of the Earth and the form of the map.

As the orbital prediction errors grow dramatically with the prediction interval [15], it is essential to determine a realistic and possible short ΔT between subsequent LEO–GMS contacts. To investigate this, let us first recall that for LEO satellites with an altitude from 300 to 1000 km, the orbital period to complete one full cycle ranges typically from 1.5 to 1.7 h, and the ground track of the orbital cycle shifts with time. Taking the satellite GRACE FO-1 [16] as an example, which has an altitude of about 500 km and an inclination of about 89°, Figure 2 shows the ascending time of the satellite above an elevation mask angle of 5° for different ground grid points on 14 August 2018, from 0:00 to 1:30 in GPS time (GPST) in panel (a), and from 6:00 to 7:30 in panel (b), as two example periods that each approximately represent a full-cycle of the satellite around the Earth. In the blank areas, the satellite is not visible above the elevation mask during the corresponding period. From Figure 2, it can be observed that in mid- and low-latitude areas, two banded areas with a longitude difference of about 167° can observe the satellite with a time difference of about 0.5 to 1 h. The visible area of the satellite, however, shifts with time. Large time gaps between subsequent satellite contacts could exist for one GMS located at a mid- or low latitude.

**Figure 3:**
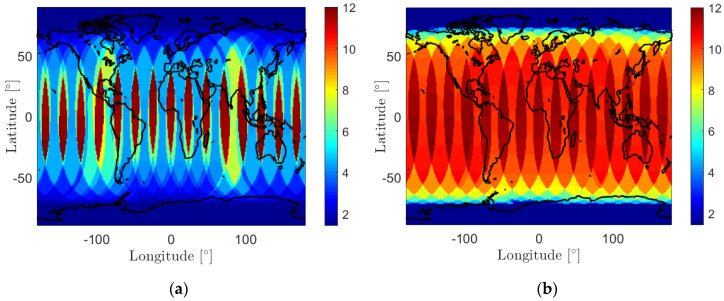
(**a**) The mean and (**b**) the maximum time gaps between subsequent satellite visible periods for GRACE FO-1 on 14 August 2018. The almost equal colors around the North and the South Poles are caused by the spherical shape of the Earth and the form of the map.

Figure 3 further illustrates this with the mean and maximum time gaps between the subsequent satellite visible periods for one full day (14 August 2018) shown in panels (a) and (b), respectively. At a high latitude of ±80°, the maximum time gap is around 1.5 h, while at a latitude of ±50° and ±20°, the value could increase to about 9.7 and 10.9 h, respectively. This implies that to enable a short update interval ΔT, for LEO satellites with high inclinations such as GRACE FO-1, one needs either to locate the GMS at high latitudes or to increase the network density.

**Figure 4:**
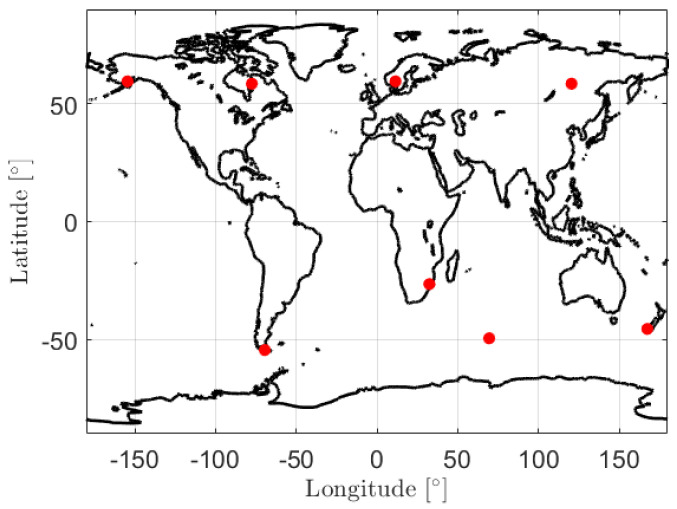
Example of the ground-based ground monitoring stations (GMSs) distributed in latitudes within $\pm 60^{{}^{\circ}}$, so that a maximum observation time gap not exceeding 1 h can be ganranteed for GRACE FO-1 on 14 August 2018. Note that the GMS located around $70^{{}^{\circ}}$ E and $50^{{}^{\circ}}$ S is above an island in the Indian Ocean.

Table 1 shows the minimum number of the land-based GMSs required to guarantee an observation gap that does not exceeding $\Delta T_{gap}$ for GRACE FO-1 on 14 August 2018. The table also gives the options to exceed the usage of stations at high latitudes outside a certain range. The stations having small continuous observation gaps and long daily observing times are selected with a high priority. From Table 1, it can be observed that for GRACE FO-1, using high-latitude GMSs is essential to reduce the number of the required GMSs for a short observation gap of—e.g., 1 h. As an example, Figure 4 gives the GMSs distributed in latitudes within ±60° to guarantee a maximum $\Delta T_{gap}$ not exceeding 1 h.

In [12], an uploading interval of 1 h is assumed. In this study, the Level A orbital positions with low accuracy are predicted up to 6 h considering the fact that different GMSs may be required for different LEO satellites, the ground infrastructure for LEO-based navigation signals needs time to be fully developed, and the high burden of the data downloading and uploading at the GMSs might become a challenge when the number of the LEO satellites becomes huge. 

#### 2.1.2. Orbit Estimation and Prediction

In this work, the reduced-dynamic orbits were processed using the Bernese GNSS Software Version 5.2 [17] combining the GPS observations and existing dynamic models. After data pre-processing and detection and exclusion of possible observation outliers, the ionosphere-free combination of the GPS phase and code observations were used to estimate the six Keplerian elements at the initial condition and certain dynamic parameters to compensate for model deficiencies. The six Keplerian elements include the semi-major axis a, the eccentricity e, the inclination I, the right ascension of the ascending node Ω, the argument of perigee ω, and the argument of latitude at the initial time $u_{0}$. The six Keplerian elements also serve as the initial state for the orbit prediction later. The estimable dynamic parameters include constants in the radial (R), along-track (S) and cross-track (W) directions ($R_{0}$, $S_{0}$, $W_{0}$) and optionally the harmonic (sine (s) and cosine (c)) periodic terms ($R_{c}$, $R_{s}$, $S_{c}$, $S_{s}$, $W_{c}$, $W_{s}$). The estimable dynamic parameters also include the stochastic velocity pulses δv at pre-defined time points or piece-wise constant accelerations δa for pre-defined time intervals. In this study, different options for the estimable dynamic parameters were tested with the stochastic dynamic parameters set for different time intervals from 15 min to 24 h (see Table 2). When estimating the stochastic accelerations δa as piece-wise constants, the periodic terms $R_{c}$, $R_{s}$, $S_{c}$, $S_{s}$, $W_{c}$, $W_{s}$ were not estimated to avoid over-parameterization [17]. Note that the stochastic parameters are constrained to zero with a standard deviation of $5\times 10^{-9}$ m/$s^{2}$ for the piece-wise constant accelerations and $5\times 10^{-6}$ m/s for the velocity pulses. To form the design matrix during the least-squares adjustment, the partial derivatives of the position vector r with respect to the orbital parameters were obtained by solving the variational equations with numerical integration [18].

With the initial condition and the dynamic parameters estimated, the orbits can be estimated and predicted by solving the equation of motion with numerical integration:

(1) $$\ddot{r}=-\mathrm{GM}\times \frac{r}{{\left|r\right|}^{3}}+a_{p},$$

where $\ddot{r}$ represents the second derivative of the position vector r with time, and |·| denotes the norm of a vector. GM represents the Earth gravitational constant. $a_{p}$ denotes the perturbation acceleration including the gravitational terms of the Earth and the other planets, different tidal effects, the general relativistic term, solar radiation pressure and air drag. Note that the latter two terms are largely absorbed by the estimable dynamic parameters in Table 2. For orbit prediction, the last set of the stochastic dynamic parameters were extended to the prediction interval and were used for the numerical integration. The processing details and the dynamic models used in the processing are summarized in Table 3. An observation sampling interval of 30 s was chosen for faster processing, where the resulted orbital accuracy should not differ much compared to the case when a shorter sampling interval of, e.g., 10 or 20 s was used [19]. The prediction here refers to the orbital extrapolation. A short prediction sampling interval of 1 s was applied to provide a higher redundancy and thus better estimation of the ephemeris parameters.

#### 2.1.3. Ephemeris Parameters

Compared to the GNSS satellites, LEO satellites are nearer to the Earth and are thus more influenced by the Earth’s gravitational field and the air drag. Furthermore, due to the almost circular orbits of the LEO satellites, singularity problems need to be considered between different ephemeris parameters. Studies have shown that when using the 16 ephemeris parameters as used in the GPS LNAV message, the fitting interval needs to be shortened to 10 to 20 min for LEO satellites to match the similar fitting errors as those for the GPS satellites with a fitting interval of 4 h [3]. It was also shown in [11] that adding additional ephemeris parameters and transforming certain ephemeris parameters are helpful to reduce the fitting errors and avoid the singularity problems. In this study, 20 ephemeris parameters were used to fit the predicted orbits within a fitting interval of 20 min. The ephemeris parameters were updated every 10 min. The 20 ephemeris parameters include (i) the rate of the semi-major axis $\dot{a}$, (ii) the mean motion rate $\dot{n}$, and (iii) and (iv) the amplitude of the two third-order harmonic correction terms to the orbit radius $C_{rs3}$ and $C_{rc3}$, in addition to the 16 traditional parameters in the GPS LNAV message. The eccentricity e, the argument of perigee ω and the mean anomaly at the reference time $M_{0}$ were transformed to $e_{x}$, $e_{y}$ and $\lambda_{0}$ to avoid the singularity problems with [11]:

(2) $$e_{x}=e\times cos\left(\omega \right),$$

(3) $$e_{y}=e\times \sin\left(\omega \right),$$

(4) $$\lambda_{0}=\omega +M_{0}.$$

The 20 ephemeris parameters are summarized in Table 4, where $t_{oe}$, $a_{0}$, Δn, $\Omega_{0}$, $I_{0}$, $\dot{I}$, $\dot{\Omega }$ represent the reference time of ephemeris, the semi-major axis at the reference time, the mean motion correction, the longitude of ascending node at the weekly epoch, the inclination at the reference time, the inclination rate and the right ascension rate, respectively. $C_{us}$, $C_{uc}$, $C_{rs}$, $C_{rc}$, $C_{is}$, $C_{ic}$ are the amplitudes of the second-order sine and cosine harmonic correction terms to the argument of latitude u, the orbit radius r, and the inclination I, respectively. 

The newly added additional parameters are connected with the GPS LNAV ephemeris parameters as follows [11]:

(5) $$a=a_{0}+\dot{a}\left(t-t_{oe}\right),$$

(6) $$n=\sqrt{\frac{\mathrm{GM}}{a^{3}}}\;+\;\Delta n+\dot{n}\left(t-t_{oe}\right),$$

(7) $$\delta r=C_{rs}\sin\left(2u\right)+C_{rc}\cos\left(2u\right)\;+\;C_{rs3}\sin\left(3u\right)+C_{rc3}\cos\left(3u\right),$$

where n and δr denote the mean motion and the orbit radius correction. 

The ephemeris parameters (except for $t_{oe}$) were then estimated in the least-squares adjustment using, for example, 1 s-predicted orbits. The partial derivatives of the orbital positions with respect to the ephemeris parameters can be referred to [11,25]. Note that the reference time of the ephemeris $t_{oe}$ was considered in the middle of each fitting interval. The estimated ephemeris parameters were then used to compute the orbital positions in the Earth-fixed coordinate system according to [26].

**Figure 5:**
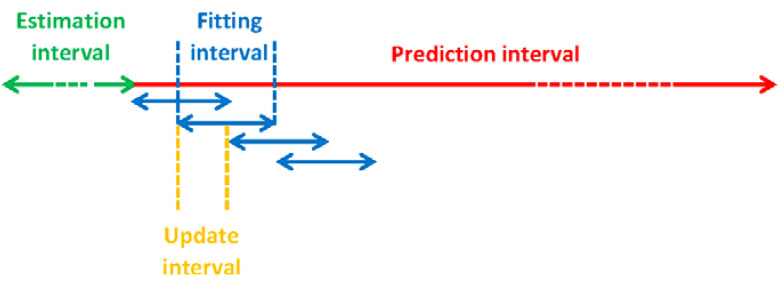
Relationships between the estimation, the prediction intervals of the orbits, the fitting and the update intervals ($\Delta t_{U}$) of the ephemeris parameters.

As the prediction interval of, e.g., 6 h is much longer than the ephemeris fitting interval of 20 min, different sets of ephemeris parameters were fitted into the orbits for the same prediction process. The relationship between the orbital prediction interval, the fitting and the update intervals of the ephemeris parameters are illustrated in Figure 5. As shown in Figure 5, the ephemeris parameters could be fitted within different fitting intervals (shown by a blue colour) using the same set of the predicted orbits of several hours (shown in red). The orbit ephemeris has a shorter update interval (shown in yellow) than the fitting interval. Note that the predicted orbits used to fit the ephemeris parameters could already deviate largely from the true orbits, where the deviation is closely dependent on the prediction interval.

#### 2.1.4. Alternative Approaches

Instead of uploading the ephemeris parameters to the LEO satellite, there are also other approaches to deliver the Level A products. As examples, it is also possible to:

- Upload the initial conditions and the estimated dynamic parameters with the numerical integration performed onboard, or directly uplink the predicted orbits to the LEO satellite [12].
- With enough computational power onboard the LEO satellites, it is also possible to make use of the GNSS broadcast ephemeris and observations, directly compute the real-time LEO orbits onboard, and extrapolate them for a short time in the future—i.e., tens of seconds [27].
- Make use of the GNSS broadcast ephemeris and observations, directly compute the SPP solutions onboard in real time, and broadcast the epoch-wise positions to the users.

In this contribution, the ephemeris parameters are proposed to be processed in the MPC and uplinked to the satellites following a similar strategy as for the GPS satellites. The advantages of applying such an approach are summarized as follows:

- No heavy burden on the LEO onboard computational power, where fast processing is carried out using high-performance computers in the MPC.
- Comprehensive dynamic models can be used for the POD.
- High-accuracy real-time GNSS products can be continuously obtained via Internet links.
- Only a limited amount of parameters (for each LEO satellite) are transferred during the uploading process.
- The navigation information can be downlinked to users with a relatively low sampling rate—e.g., 10 min.
- The LEO orbits derived from the broadcast ephemeris are smooth, which is suitable for the polynomial fitting of the precise Level B orbits (see Section 2.2).

At the same time, certain challenges exist for the LEO POD following the proposed approach. They are summarized as follows:

- Multiple GMSs might be needed to guarantee the upload intervals of several hours.
- With the rapidly increasing number of the LEO satellites, a heavy burden is put on the downlink and uplink systems at the GMSs.
- A high-grade GNSS receiver is required onboard the LEO satellite.

### 2.2. Level B Products

The Level B products refer to the high-accuracy orbital products—i.e., with an accuracy of centimeters. As shown in [4], one of the major advantages of LEO-based positioning is the rapidly changing satellite geometry, which will lead to a fast convergence time in the PPP. High-accuracy LEO orbital products with an accuracy of centimeters, here the Level B products, are essential for realizing these benefits. To achieve the required high orbital accuracy, the LEO Level B products were assumed to be processed on the ground using high-accuracy real-time GNSS orbits and clocks as explained in Section 2.1.2. Compared to the Level A products, the Level B products were predicted only for a short period into the future, and were fitted into a low-order polynomial as corrections to the broadcast ephemeris (Section 2.1), expressed as:

(8) $$r_{B}-r_{A}=p_{0}+p_{1}(t_{i}-t_{0})+\cdots +p_{m}{\left(t_{i}-t_{0}\right)}^{m},$$

where $r_{A}$ and $r_{B}$ represent the Level A orbits computed from the broadcast ephemeris, and the short-term predicted precise orbits as mentioned above, respectively. $p_{k}$ (k=0,⋯, m) are the 3D polynomial coefficients with m denoting the highest polynomial degree. $t_{0}$ and $t_{i}$ represent the correction reference time and the fitting time, respectively. Note that the dynamic model used for predicting the short-term precise orbits $r_{B}$ is based on the existing models in Table 3 and the best option for short-term orbit prediction in Table 2, which will be discussed in Section 3.

**Figure 6:**
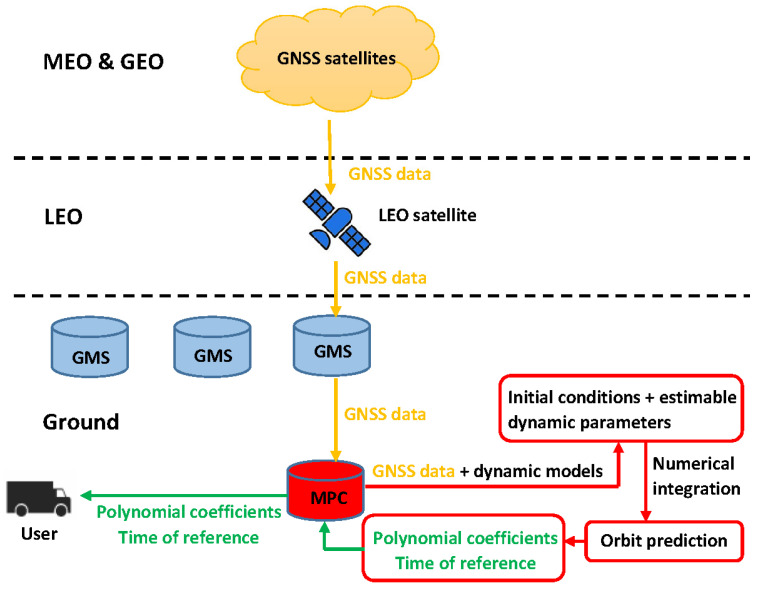
Process of generating the Level B orbital products.

According to [14], the IGS RTS orbital parameters are fitted to the predicted orbits with a prediction interval of 60 s considering the latency caused by data collection, processing and combination, although the actual time interval between the correction reference epoch and the user processing epoch could be longer due to the sampling interval of the corrections. In this study, the same interval of 60 s was applied for the orbit prediction and polynomial fitting [28]. With the GNSS observations collected and downlinked by the LEO satellite, the MPC is supposed to collect the information from the GMS, process the precise orbits, and produce the polynomial coefficients to bridge the precise orbits and the Level A products. The polynomial parameters and the corresponding reference time are then transmitted to users with a relatively high sampling rate—e.g., 10 s, via Internet links. The process for generating the Level B products is illustrated in Figure 6. The details of the Level B products are summarized in Table 5.

#### Alternative Approaches

The advantages of the proposed approach can be summarized as follows:

- No heavy burden on the LEO onboard computational power; comprehensive dynamic models can be used for the POD; fast processing with high-performance computers.
- High-accuracy real-time GNSS products can be continuously obtained with Internet links free of charge.

**Figure 7:**
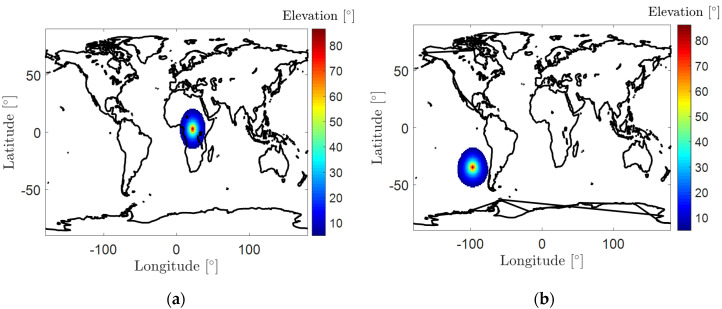
Areas observing GRACE FO-1 at (**a**) 4:15:00 and (**b**) 0:10:00 on 14 August 2018, with an elevation mask angle of 5°.

At the same time, to realize the approach, a dense network is needed to view the LEO satellite all the time due to their small footprints. This could be a high requirement on the ground infrastructure. Taking the periods when GRACE FO-1 has low-latitude footprints as an example, at least one ground station is needed within circular areas with a radius of about 17.5° in latitude and longitude (see Figure 7a). Sometimes this is hardly feasible for periods when the LEO footprints are above the ocean. Figure 7b shows an example of the area that observes GRACE FO-1 at 00:10:00 on 14 August 2018, above the elevation mask angle of 5°. The footprint is generally above the Pacific Ocean and is difficult to be observed by any ground-based monitoring stations. As such, for a certain time epoch, the ground-processed high-accuracy orbital products might not be available for certain LEO satellites in real time. The near-real-time orbits with a latency of a few hours can be delivered to users instead, which have a reduced accuracy due to the longer orbit prediction interval. These missing real-time orbits could be relevant to marine users, but not the land users who cannot observe these missing LEO satellites. Similarly, in remote areas without a lot of users, a low density of network can be accepted as the corresponding real-time Level B orbits are not heavily required there. It is worth noting that even when a dense network is available for the generation of the Level B products, the Level A products are still useful for users that do not have high requirements for the positioning accuracy, for users of relative positioning over short baselines, for users without the need of an Internet link, or without the capability to receive or process high-sampled corrections.

Instead of generating the Level B products in the MPC, one can also consider producing the precise orbits onboard, provided that precise GNSS products broadcast from higher satellites can be continuously received by the LEO satellites and enough computational power is available onboard. Simulations have been performed for an on-orbit real-time POD scenario using the Fugro precise GNSS products broadcast by GEO satellites, where a sub-decimeter accuracy can generally be achieved and the data gaps at high latitudes could increase the 3D RMSE to 8.5 cm [29]. In addition to the commercial services, high-precision GNSS products are nowadays also directly broadcast by navigation satellites from the Japanese Quasi-Zenith Satellite System (QZSS) within, e.g., the Multi-GNSS Advanced Demonstration of Orbit and Clock Analysis (MADOCA) service and the centimeter level augmentation service (CLAS) [30]. Another example is the new generation of the satellite-based augmentation system (SBAS) initiated by Australia and New Zealand, which broadcasted high-precision PPP corrections via L1 and L5 to the GPS and GPS/Galileo users, respectively, within the test bed [31,32]. Although the current service areas of the QZSS- and the SBAS-based PPP corrections are still limited to the Asia-Pacific region, with the increase in the number of new-generation SBAS services (expected for the Wide Area Augmentation System (WAAS) and the European Geostationary Navigation Overlay Service (EGNOS), etc.), a bright future is expected for the real-time onboard LEO POD with an accuracy of a few centimeters.

## 3. Test Results

In this section, the orbital errors of the Level A and Level B products are analyzed using a typical LEO satellite GRACE FO-1 from 14 to 20 August 2018. GRACE FO-1 has a low orbital height of about 500 km in the test period. It suffers from large influences of the Earth’s gravitational field and the air drag and is subject to relatively large fitting errors of the ephemeris parameters due to its low altitude [11]. For the validation of the orbital errors, the precise orbits provided by the Jet Propulsion Laboratory (JPL) are used as the reference [33]. The real-time precise GPS orbits and clocks were obtained from the National Centre for Space Studies (CNES, France) [20]. The orbital errors are analyzed in terms of the 3D root mean square error (RMSE) and the orbital user range error (OURE) as follows:

(9) $$\mathrm{OURE}=\sqrt{w_{R}^{2}\sigma_{R}^{2}+w_{SW}^{2}\left(\sigma_{S}^{2}+\sigma_{W}^{2}\right)},$$

where $\sigma_{R}$, $\sigma_{S}$ and $\sigma_{W}$ represent the RMS of the orbital errors in the radial, along-track and cross-track directions, respectively. The coefficients $w_{R}$ and $w_{SW}$ are related to the orbital height [34], and amount to about 0.457 and 0.629 for GRACE FO-1 during the test period. The orbital height amounts to about 503 km, which is approximated using the JPL reference orbits over the test period and the Earth’s radius, 6371 km. 

### 3.1. Level A Products

As described in Section 2.1, the reduced-dynamic orbits are estimated within a 24 h time interval and are predicted for 6 h with different dynamic models. This section shows the prediction errors using GRACE FO-1 data from 14 to 20 August 2018. The starting time of the estimation is shifted by 1 h for each 30 h data sample—i.e., the first data sample starts at 0:00 on 14 August 2018, and the last sample starts at 18:00 on 19 August 2018. In total, 139 data samples were used for the tests. The ephemeris parameters were fitted to the predicted orbits within 20 min intervals and updated every 10 min. 

**Figure 8:**
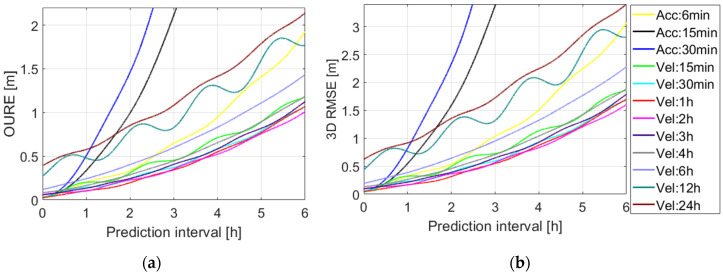
(**a**) The orbital user range error (OURE) and (**b**) the 3D root mean square error (RMSE) of the predicted orbits applying different dynamic models (Table 2).

The OURE and the 3D RMSE of the predicted orbits are shown in Figure 8 applying different dynamic models. The legends started with “Acc” represent the cases in which piece-wise constant accelerations were estimated within a pre-defined interval (Option A in Table 2), and those starting with “Vel” represent the cases in which stochastic velocity pulses were set up at epochs separated by a pre-defined interval (Option B in Table 2) [17]. For the former cases, the three constant dynamic terms $R_{0}$, $S_{0}$, $W_{0}$ and the six Keplerian elements at the initial condition were also estimated, while for the latter cases the periodic terms $R_{c}$, $R_{s}$, $S_{c}$, $S_{s}$, $W_{c}$, $W_{s}$ were estimated in addition. The stochastic parameters were used to compensate for the effects that are not included in the existing orbital model (Table 2). Different sets of the stochastic parameters have different compensation effects. This explains the fluctuations of the OUREs and 3D RMSE with different amplitudes and a period of about 1.5 h. From Figure 8, it can be observed that both the OURE and the RMSE grow with the prediction time. For prediction periods longer than 0.5 h, estimating the stochastic velocity pulses every 1 (red lines) or 2 h (magenta lines) generally provides the lowest prediction errors among all the tested options. For a long-term prediction, with a prediction interval longer than 4 h, the option “Vel: 2h” (magenta lines) offers the lowest OURE and 3D RMSE. 

**Figure 9:**
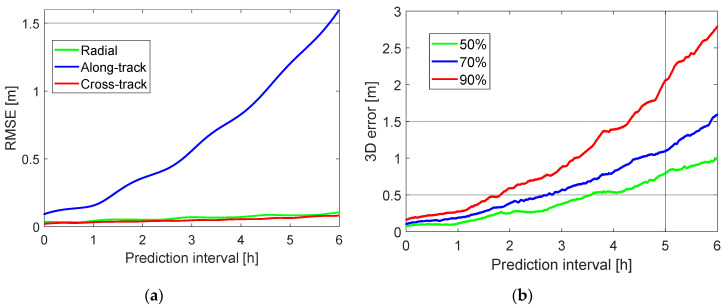
(**a**) RMSE of the predicted orbits in the radial, along-track and cross-track directions and (**b**) the 50%, 70% and 90% percentile lines of the 3D prediction errors. The dynamic model used applies stochastic velocity pulses set up for every 2 h (Table 2).

Considering the option that stochastic velocity pulses were set up for every 2 h, the budget of the prediction errors is given for different prediction intervals in Table 6. It can be observed that the along-track prediction errors play the dominant role among the three orbital directions (see also Figure 9a). For the prediction intervals of 1 and 2 h, the OUREs of about 0.1 and 0.2 m using the proposed dynamic model are smaller than those shown in [12]—about 0.3 and 0.5 m without using additional accelerometers onboard. For a long prediction interval of 6 h, the OURE amounts to about 1 m. The 50%, 70% and 90% percentile lines of the 3D prediction errors are further illustrated in Figure 9b. It can be observed that 90% of the 3D prediction errors are within 0.3 m at the prediction interval of 1 h, and the value increases to about 2.8 m for a prediction interval of 6 h.

**Figure 10:**
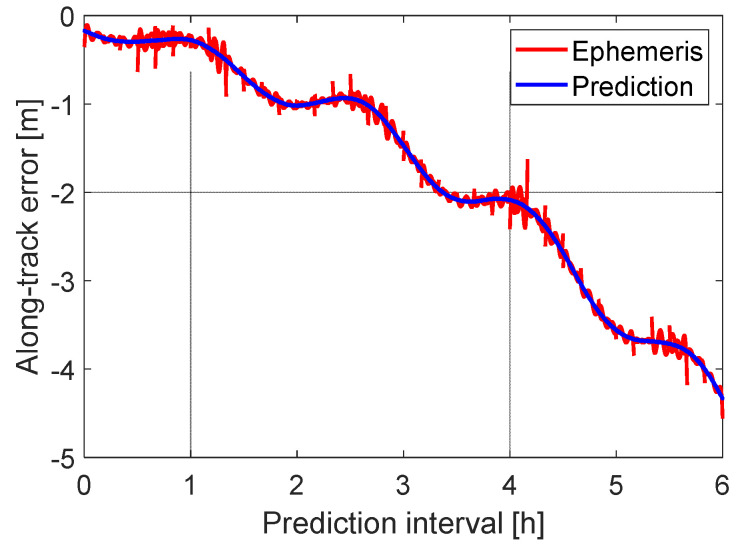
The along-track errors of the predicted and the ephemeris orbits from 23:00 on 15 August 2018, to 5:00 on 16 August 2018.

Fitting the LEO-specific ephemeris parameters (Section 2.1.3) to the predicted orbits within 20 min, additional fitting errors need to be considered in the error budget of the Level A orbital products. Taking the data sample with the largest prediction error at 6 h as an example—i.e., the data sample with the prediction interval from 23:00 on 15 August to 5:00 on 16 August—the prediction (blue) and the ephemeris errors (red) are shown in Figure 10 for the dominant along-track direction. As previously mentioned, the ephemeris parameters were obtained from a least-squares adjustment within each 20 min fitting interval, and the orbits derived from the ephemeris parameters were compared with the JPL reference orbits to form the ephemeris orbital errors (red). Each set of the ephemeris orbits were fitted within a 20 min interval and updated every 10 min. An overlapping interval of 10 min between subsequent sets of the ephemeris orbits is also included in the plot (red lines). From Figure 10 it can be observed that, compared to the prediction errors that can reach several meters, the ephemeris fitting errors—i.e., the differences between the red and the blue lines in Figure 10—are small. 

**Figure 11:**
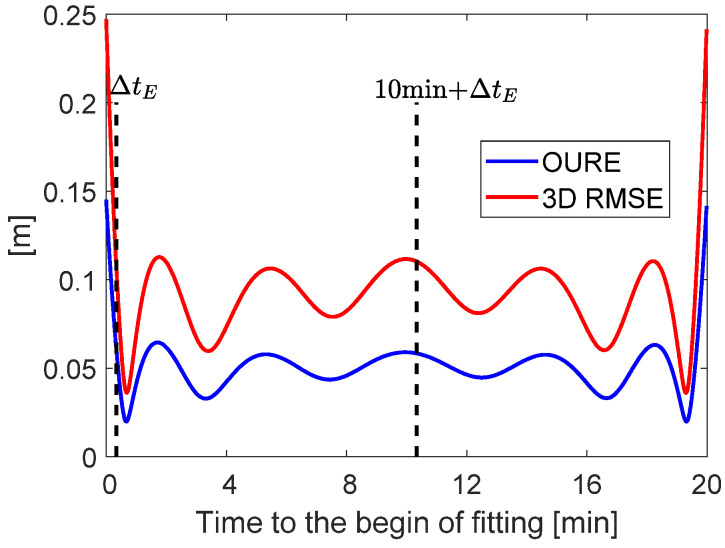
The OURE and the 3D RMSE of the ephemeris fitting errors within a 20-min fitting interval using 20 ephemeris parameters. The dashed lines mark the time interval in which the ephemeris is used by the users.

To have a closer look at the fitting errors themselves within the 20 min fitting interval, the OURE and the 3D RMSE of the fitting errors are plotted in Figure 11 using all the tested data samples. It can be observed that the OURE (blue line) is generally around 5 cm for GRACE FO-1 with an altitude of about 500 km, which is consistent with the results shown in [11]. The 3D RMSE is around 1 dm. From Figure 11 it can also be observed that border effects exist in the fitted orbits, which are reflected in the larger fitting errors at the beginning and the end of the fitting interval. This is caused by the ephemeris parameterization and the form of the design matrix in the least-squares adjustment. Similar border effects are also pronounced in the reduced-dynamic orbits of short arcs [35]. These border effects, however, should not significantly influence the POD for the users. On the one side, the ephemeris parameters are supposed to be updated every 10 min, so that the ephemeris at the end of the fitting curve is not used when a new ephemeris is available. On the other side, a minimum latency $∆t_{E}$ could be allowed between the transmission time of the ephemeris and the beginning of the fitting interval—e.g., for the GPS satellites on 8 July 2018, a latency of 18 s can mostly be observed. As such, and as shown by the dashed lines in Figure 11, assuming a $∆t_{E}$ of 20 s, the set of the ephemeris is actually used by the users with the time interval from $∆t_{E}$ to 10 min + $∆t_{E}$.

**Figure 12:**
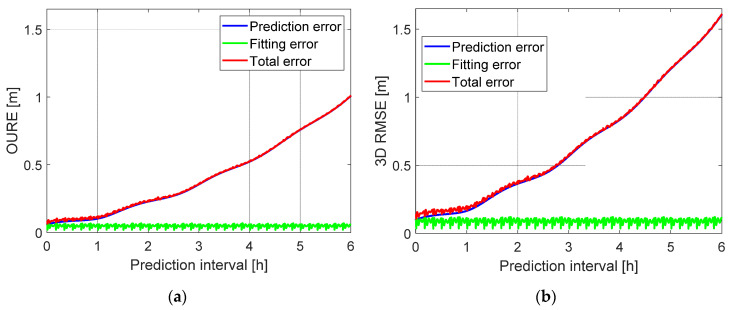
(**a**) The OURE and (**b**) the 3D RMSE of the prediction errors, the fitting errors and the total errors of the Level A orbital products.

Using the ephemeris between $∆t_{E}$ and 10 min + $∆t_{E}$ for each set of the ephemeris parameters, the error budget of the Level A orbital products is illustrated in Figure 12 using all the tested data samples. It can be observed that the prediction errors (blue lines) play the dominant role in the Level A products, while the fitting errors (green lines) have only a slight influence on the short-term prediction, and almost no influence on the long-term prediction (see the overlapped blue and red lines for the long-term prediction). For the fitting errors (green lines), systematic effects can be observed with a period of 10 min, which more or less match the pattern between the dashed lines in Figure 11. The fitting errors do not vary with the prediction time and have a mean OURE and a mean 3D RMSE of about 5 and 9 cm, respectively. The error budget is further shown in Table 7 for different prediction intervals. For a prediction interval of 1, 2 and 6 h, the total ephemeris OURE amounts to about 0.1, 0.2 and 1 m, respectively.

### 3.2. Level B Products

The same data sets as used in Section 3.1 are used to test the Level B products in this section. Different from the Level A products, in the Level B products, the orbits were extrapolated only for a short period of tens of seconds, and the differences from the Level A orbits were then fitted using a low-order polynomial. Taking the IGS RTS orbital products as an example, this prediction and fitting interval amounts to 60 s and the predicted orbits are fitted with a linear polynomial [14]. In this contribution, the same prediction and polynomial fitting interval of 60 s is applied, and tested for one-, two- and three-degree polynomials. 

**Figure 13:**
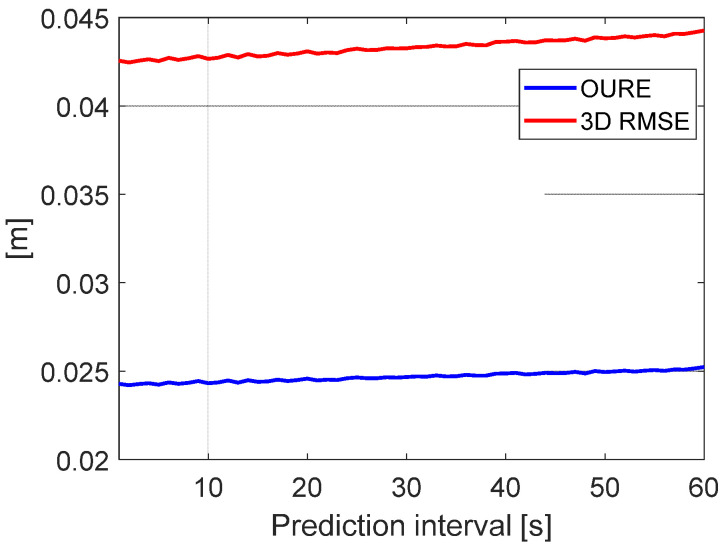
The OURE and the 3D RMSE of the predicted orbits for the short-term prediction.

Using the option “Acc:6min” in the dynamic model, which is the most suitable option for the short-term orbit prediction (see Figure 8), the OURE and the 3D RMSE are shown in Figure 13 for the orbit prediction of 60 s. It is shown that the errors slightly increase with the increase in the prediction interval, but the growth is very limited, at a few millimeters, for such a short period. The general OURE and 3D RMSE are at the level of a few centimeters, which fulfils the accuracy requirement of the Level B orbital products. Note that the prediction interval of 60 s was used in this study as an example for the purpose of demonstration. The results show that by using the same dynamic model, the short-term prediction of the LEO orbits within 10 min can all reach good accuracy—i.e., with an OURE within 4 cm. To reduce the computational time, super-computers and Kalman filter can be used for producing Level B products in the future.

**Figure 14:**
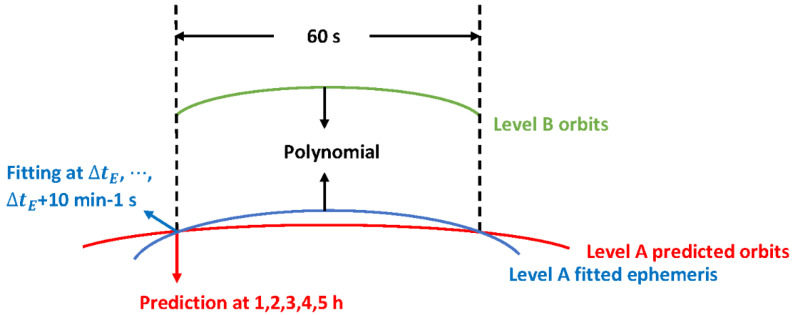
Test strategy for the low-order polynomial corrections merging the Level A and Level B orbits.

The differences between the Level A broadcast orbits and the precise orbits predicted over short periods are described by a low-order polynomial (Equation (8)). As the polynomial fitting errors are associated with the short-term behaviors of both the precise orbits and the Level A orbits at different phases, tests were performed where the beginning of the polynomial fitting corresponded to a different prediction time of the Level A orbits, from 1 to 5 h, and to a different ephemeris fitting time, from $∆t_{E}$ to $∆t_{E}$+10 min^−1^ s (see Figure 11) with a sampling interval of 30 s (see Figure 14). Within the 60 s polynomial fitting interval, one consistent set of the ephemeris parameters was used for the Level A orbits.

**Figure 15:**
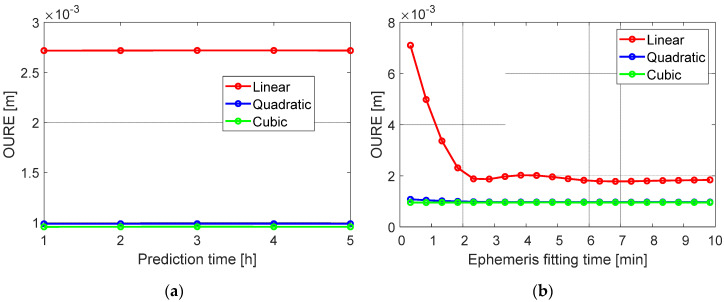
The OUREs of the polynomial fitting errors when the beginning of the fitting interval corresponds to (**a**) different prediction times and (**b**) different ephemeris fitting times of the Level A products.

The OUREs of the fitting errors are shown in Figure 15 where the start of the fitting interval corresponds to different orbit prediction times and ephemeris fitting times of the Level A products. 134 samples of the 60 s predicted orbits were used for the tests, where each of the 60 s precise orbits are differenced with the Level A orbits at five different prediction periods and 20 different ephemeris fitting periods (see Figure 14), amounting to 13,400 data samples used for the polynomial fitting in total. From Figure 15, it can be observed that, in general, the fitting errors are small—i.e., at several millimeters. The quadratic polynomials match the orbital differences better than the linear polynomials, and increasing the polynomial degree from two to three almost led to no further benefits. The fitting errors do not vary much with the prediction times of the Level A orbits, but do show some differences at different ephemeris fitting times. A variation in quadratic form is pronounced within the 60 s polynomial fitting period when the start of the fitting interval corresponds to the first 2 min of the ephemeris fitting. This leads to several millimeters of degradation when a linear polynomial is applied. 

**Figure 16:**
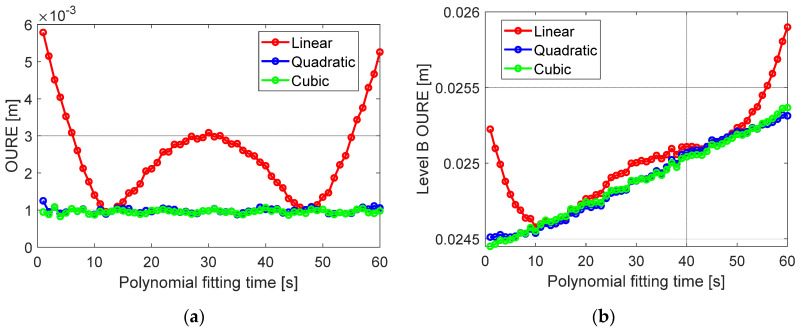
The OURE of (**a**) the polynomial fitting errors and (**b**) the total Level B orbital errors concerning the polynomial fitting time.

The variation of the fitting OUREs is further illustrated in Figure 16a concerning the polynomial fitting time. It is clearly shown that applying a linear polynomial would lead to large fitting errors at the beginning, the middle and the end of the 60 s time interval, which suggests again the quadratic behavior of the orbital corrections. The degradation caused by using the linear polynomial is also significant in the total orbital errors at Level B, as shown in Figure 16b. Increasing the polynomial degree from two (blue) to three (green) leads to almost no benefits. 

The total error budget of the Level B orbital products are given in Table 8 at the beginning and the end of the polynomial fitting. It can be observed that the prediction errors still play a major role in the total error budget, while increasing the polynomial degree from one to two can reduce the fitting OUREs by a few millimeters. Further increasing the polynomial degree does not lead to obvious benefits in fitting the OUREs. In general, the Level B orbits have an accuracy of a few centimeters and can fulfil the requirement for single-receiver high-precision positioning service. Increasing the polynomial degree from one to two or three does not have significant influences on the total error budget of the Level B products.

## 4. Conclusions

With thousands of LEO satellites launched or planned to be launched in the near future, studies have been performed to utilize the LEO signals for navigation purposes. The increased satellite number with their improved signal strength, compared to GNSSs, and the rapidly changing geometry of the LEO mega-constellations, are shown to be beneficial to the positioning service on the ground. 

One main condition to realize the LEO-based positioning is the knowledge of the orbital positions of the LEO satellites. Relying on the GNSS observations collected onboard the LEO satellites, this study proposes two procedures to provide the LEO orbital products. The first is with a relatively low accuracy at meter-level, i.e., the Level A products, and the second is high-accuracy LEO orbital products at centimeters—i.e., the Level B products. The orbits are proposed to be processed on the ground with good dynamic models, extrapolated to up to several hours and tens of seconds for the Level A and B products, respectively. For the Level A products, the long-term predicted orbits are supposed to be fitted into subsequent sets of LEO-specific ephemeris parameters, uplinked to the LEO satellites and then broadcast to users. Based on the broadcast ephemeris, low-order polynomials are generated to merge the differences between the Level A orbits and the short-term predicted precise orbits. The polynomial corrections and the time of reference, as the Level B products, are then transmitted to users via Internet links to achieve high orbital accuracy of centimeters.

In this study, the real data of a typical LEO satellite, GRACE FO-1, which resembles other LEO satellites that can be used for positioning, with a relatively low altitude of about 500 km, were used for the tests. For the Level A products, it was found that the prediction errors play the dominant role in the total error budget, and the prediction interval and the corresponding dynamic models used are the key factors to keep the prediction errors at an acceptable level. The LEO orbital configuration, the latitude and the network density of the ground monitoring stations are the limiting factors for the maximum time gaps between subsequent satellite contacts, and thus the limiting factors for the required orbital prediction period. In this study, a prediction interval of up to 6 h is proposed. With different options of the estimable dynamic parameters tested, it was found that estimating the stochastic velocity pulses every 2 h in addition to the Keplerian elements at the initial condition, as well as the constant and periodic accelerations, provides the best results for mid- and long-term prediction. The OURE of the Level A orbits amount to about 0.1, 0.2 and 1 m for prediction intervals of 1, 2 and 6 h, respectively. The ephemeris fitting errors are only around several centimeters and do not have significant influences in cases of the mid- and the long-term prediction.

The Level B products are extrapolated for a much shorter period—i.e., 60 s—in this study and the OURE of the total errors amounts to several centimeters. The prediction errors still play a major role in the total error budget. At the same time, it was found that increasing the polynomial degree from one to two could reduce the fitting OUREs by a few millimeters, while further increasing the degree value to three brings almost no benefits. In general, the Level B orbits could reach an accuracy of a few centimeters, and increasing the polynomial degree from one to two or three does not significantly influence the total error budget of the Level B products.

In summary, after overcoming the challenges related to the hardware infrastructure, the LEO orbital products can theoretically be provided to users with a sub-meter to meter-level accuracy in the Level A products as broadcast ephemeris, and with an accuracy of centimeters in the Level B products as high-accuracy high-rate corrections. 

## Figures and Tables

**Table 1:** Minumum number of the land-based GMSs required to guarantee an observation gap not exceeding $\Delta T_{gap}$. The data of GRACE FO-1 on 14 August 2018, were used for the calculation.

Latitude (*B*) Range	$\;\;\Delta T_{gap}\;=\;1\;h$	$\;\;\Delta T_{gap}\;=\;2\;h$	$\;\;\Delta T_{gap}\;=\;3\;h$	$\;\;\Delta T_{gap}\;=\;6\;h$
|B|≤60°	8	5	2	2
|B|≤70°	5	2	2	1
|B|≤80°	2	1	1	1
All	2	1	1	1

**Table 2:** Options for estimable parameters for orbit estimation and prediction.

Category	Parameters		Estimation Interval
Keplerian elements	a, e, I, Ω, ω, $u_{0}$		24 h
Dynamic parameters	Option A	$R_{0}$, $S_{0}$, $W_{0}$ δa	24 h 6/15/30 min
	Option B	$R_{0}$, $S_{0}$, $W_{0}$, $R_{c}$, $R_{s}$, $S_{c}$, $S_{s}$, $W_{c}$, $W_{s}$ δv	24 h 15/30 min, 1/2/3/4/6/12/24 h

**Table 3:** Processing parameters and the dynamic models used in the processing. The following abbreviations are used: CNES for the National Centre for Space Studies in France, JPL for the Jet Propulsion Laboratory in the USA, and IERS for the International Earth Rotation and Reference Systems Service.

Parameters/Models	Details
Observations	GPS IF combination (L1/L2), code + phase
Sampling interval	Observations: 30 s; Prediction: 1 s
Estimation interval	24 h
Prediction interval	6 h
Elevation mask	5°
GPS orbits/clocks	CNES real-time products [20]
Dynamic models	Earth gravity terms: EGM2008 (degree: 120) [21]
	Gravity terms of other planets: JPL DE405 [22]
	Solid Earth tides, Pole tides: IERS 2010 [23]
	Ocean tides: FES2004 [24]
	General relativistic effects

**Table 4:** Ephemeris parameters for low Earth orbit (LEO) satellites.

Category	Ephemeris Parameters
GPS LNAV ephemeris parameters	$t_{oe}$, $\sqrt{a_{0}}$, Δn, $\Omega_{0}$, $I_{0}$, $\dot{I}$, $\;\dot{\Omega }\;$, $C_{us}$, $C_{uc}$, $C_{rs}$, $C_{rc}$, $C_{is}$, $C_{ic}$
Transformed GPS LNAV ephemeris parameters	$e_{x}$, $e_{y}$, $\lambda_{0}$
Additional ephemeris parameters	$\dot{a}$, $\dot{n}$, $C_{rs3}$, $C_{rc3}$

**Table 5:** Details of the Level B orbital products.

Parameters/Models	Details
Strategy of orbit estimation	Reduced-dynamic orbits (Section 2.1.2)
Sampling interval of the observations	30 s
Strategy of orbit prediction	Dynamic orbits (Table 2 and Table 3)
Prediction sampling interval	1 s
Prediction interval	60 s
Polynomial degree	1, 2, 3

**Table 6:** The prediction error budget for different prediction intervals when using the dynamic model having stochastic velocity pulses set up for every 2 h (Table 2).

Prediction Interval	RMSE Radial (m)	RMSE Along-Track (m)	RMSE Cross-Track (m)	3D RMSE (m)	OURE (m)
0.5 h	0.035	0.131	0.028	0.138	0.086
1 h	0.042	0.156	0.033	0.165	0.102
2 h	0.052	0.359	0.039	0.365	0.228
3 h	0.070	0.560	0.047	0.566	0.355
4 h	0.072	0.829	0.055	0.834	0.523
5 h	0.083	1.203	0.064	1.207	0.758
6 h	0.105	1.599	0.082	1.605	1.008

**Table 7:** The error budget of the Level A products at different prediction periods.

Prediction Interval	OURE (m)			3D RMSE (m)		
	Prediction	Fitting	Total	Prediction	Fitting	Total
0.5 h	0.086	0.059	0.101	0.138	0.111	0.173
1 h	0.102	0.058	0.118	0.165	0.112	0.200
2 h	0.228	0.059	0.236	0.365	0.113	0.383
3 h	0.355	0.059	0.360	0.566	0.102	0.579
4 h	0.523	0.059	0.527	0.834	0.114	0.842
5 h	0.758	0.059	0.760	1.207	0.114	1.213
6 h	1.008	0.060	1.010	1.605	0.114	1.609

**Table 8:** Total error budget of the Level B orbital products. The results at the prediction time of 1 and 60 s are separated by “/”.

Fitting Type	Prediction Errors [cm]		Fitting Errors [cm]		Total Errors [cm]	
	OURE	3D RMSE	OURE	3D RMSE	OURE	3D RMSE
Linear polynomial	2.4/2.5	4.3/4.4	0.6/0.5	1.0/0.9	2.52/2.59	4.43/4.56
Quadratic polynomial			0.1/0.1	0.2/0.2	2.45/2.53	4.29/4.44
Cubic polynomial			0.1/0.1	0.2/0.2	2.45/2.54	4.28/4.45
